# Face Your Fears: Cleaning Gobies Inspect Predators despite Being Stressed by Them

**DOI:** 10.1371/journal.pone.0039781

**Published:** 2012-06-27

**Authors:** Marta C. Soares, Redouan Bshary, Sónia C. Cardoso, Isabelle M. Côté, Rui F. Oliveira

**Affiliations:** 1 Unidade de Investigação em Eco-Etologia, ISPA-Instituto Universitário, Lisboa, Portugal; 2 Université de Neuchâtel, Institut de Zoologie, Eco-Ethologie, Neuchâtel Switzerland; 3 Department of Biological Sciences, Simon Fraser University, Burnaby, British Columbia, Canada; 4 Champalimaud Neuroscience Programme, Instituto Gulbenkian de Ciência, Rua da Quinta Grande, Oeiras, Portugal; University of Otago, New Zealand

## Abstract

Social stressors typically elicit two distinct behavioural responses in vertebrates: an active response (i.e., “fight or flight”) or behavioural inhibition (i.e., freezing). Here, we report an interesting exception to this dichotomy in a Caribbean cleaner fish, which interacts with a wide variety of reef fish clients, including predatory species. Cleaning gobies appraise predatory clients as potential threat and become stressed in their presence, as evidenced by their higher cortisol levels when exposed to predatory rather than to non-predatory clients. Nevertheless, cleaning gobies neither flee nor freeze in response to dangerous clients but instead approach predators faster (both in captivity and in the wild), and interact longer with these clients than with non-predatory clients (in the wild). We hypothesise that cleaners interrupt the potentially harmful physiological consequences elicited by predatory clients by becoming increasingly proactive and by reducing the time elapsed between client approach and the start of the interaction process. The activation of a stress response may therefore also be responsible for the longer cleaning service provided by these cleaners to predatory clients in the wild. Future experimental studies may reveal similar patterns in other social vertebrate species when, for instance, individuals approach an opponent for reconciliation after a conflict.

## Introduction

Animals are continuously faced with a wide range of environmental and social pressures, and are forced to make decisions in order to survive [1]. Predation risk is one the most significant selective forces shaping animal behavioural strategies due to its implications for individual survival [2]. Its influence drives changes in morphology, coloration and chemical defenses, habitat use, vigilance behaviour, and even sociality across evolutionary time [3]–[5]. However, while it is clear that predation risk can induce significant changes to a prey’s behaviour and even to population dynamics, less is known about the relevant causal mechanisms that are directly responsible for these alterations, particularly when predation risk affects prey foraging decisions [6]–[7].

Predators are notorious inducers of stress responses [3]. These responses usually involve a suite of hormones known to mediate stress responses [8]. These hormones belong to two endocrine systems: the catecholamine response and the glucocorticoid response [9]). Unlike most catecholamines, glucocorticoids can cross the blood-brain barrier and access receptors in several brain regions. This makes their potential role in stress response important because in order to affect behaviour, the mediation of stress must also affect the brain [8]. Stress responses are usually characterized physiologically by the activation of the hypothalamic-pituitary-interrenal tissue axis (HPI), which leads to an increase in corticosteroid production. When referring to social stress in vertebrates, this physiological cascade is usually described as being able to elicit one of two alternative behavioural responses: a proactive response (active coping, or ‘fight-flight’) or a reactive response (passive coping, or ‘conservation-withdrawal’) [10]. Differences in coping behaviour are commonly associated with distinct hormonal responses: pro-activity presumes high sympathetic reactivity and low HPI activity, whereas reactivity is associated with low sympathetic reactivity and high HPI activity. The threshold at which the shift occurs from a more passive to an active response to a certain stimulus should be determined by individual cognitive appraisal of the stimulus [11].

Given that predators usually evoke significant behavioural responses, such as flight or freezing, from their potential prey, it is surprising that some cleanerfish readily approach predators. Some will even enter and inspect the mouth of predatory clients [12], [13]. Although predation on cleaners engaged in cleaning interactions has never been observed (reviewed by [14]), the risk of predation by carnivorous fishes is real. Predation on cleanerfish away from cleaning arenas has been observed (e.g. [15]), and various species of cleaners have been recorded in the stomach contents of predators that could have been clients ([14]). Several aspects of cleaner behavior are also consistent with the idea of risk minimization, such as the tendency to inspect predominantly safe areas such as the tail and fins of dangerous clients ([15]–[17]. It therefore becomes of interest in the context of the behavioural dichotomy in stress response described above to examine how predators affect cleanerfish stress levels, given that fleeing or freezing would prevent cleaners from engaging in interactions with these clients.

We examine this potential conundrum using cleaning gobies of the genus *Elacatinus*. Cleaning gobies are the most specialized and ubiquitous cleaners in the western tropical Atlantic. They interact regularly with a range of potentially dangerous clients [18]–[20], [12] that visit their territories (known as cleaning stations), and they do so more often than all other cleaner species in the region [20]. Although predation on cleaning gobies has never been recorded in the field, it has been observed on captive gobies [18], [21], suggesting that they are not immune to predation under non-cleaning circumstances. The behaviour of cleaning gobies towards predatory clients is puzzling. Cleaners initiate interactions with piscivores almost as soon as the latter arrive at cleaning stations, despite the fact that these clients offer no obvious foraging advantage since predators and non-predatory clients are equally infested by ectoparasites [12]. Moreover, cleaning gobies cheat, by taking mucus and scales instead of parasites, almost as often towards predators as towards non-predatory clients [12]. This behaviour contrasts with that of the Indo-Pacific bluestreak cleaner wrasse *Labroides dimidiatus*, which exhibits unconditional honesty towards predators, probably to minimise the risk of being attacked [13], [22]. Recently, Bshary and colleagues [23] proposed that short-term stress might contribute to the higher cleaning service quality provided by cleaner wrasses to predators, although no empirical endocrine evidence was provided.

Here, we investigate whether cleaning gobies perceive predatory clients as a risk by assessing their physiological stress levels when approaching and interacting with these clients. To do so, we compare the concentrations of cortisol – the main corticosteroid released by teleosts [24] – found in water holding cleaning gobies that were in visual contact with either a predator or a harmless (i.e. non-predatory) client. We then relate cortisol level to the behaviour of captive cleaning gobies towards both client types. Finally, we compared our captivity data to field observations of interactions between cleaning gobies and their client fish (predatory and harmless).

## Results

### Assay validation

**Figure 1:**
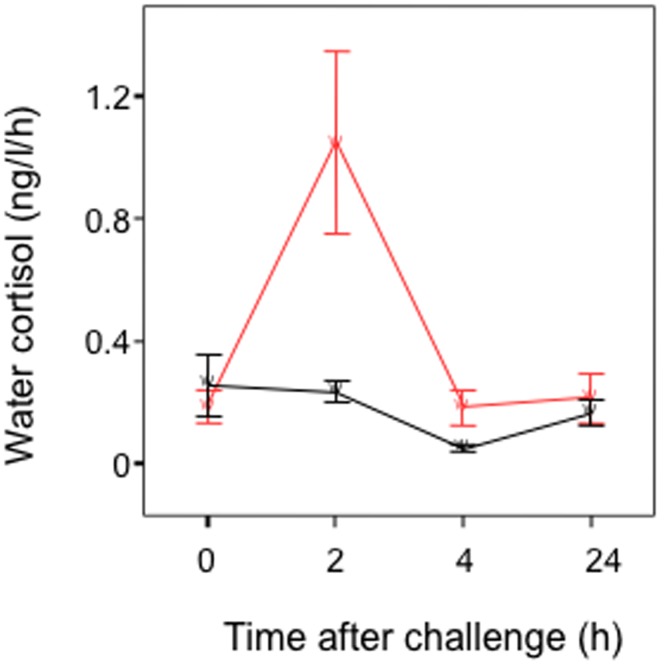
Temporal variation in cortisol levels in holding-water of individual cleaning gobies challenged with an intra-peritoneal injection of porcine ACTH (red line) or saline solution (black line). Means are shown ±1 SEM. *n* = 4 for each time period.

In trials to validate the hormone assay, cortisol immunoreactivity in holding water varied significantly over time (2-way RM-ANOVA, *F*_3, 15_ = 7.12, *P* = 0.003; Figure 1), but did not vary overall between treatments (cleaning gobies injected with ACTH versus injected with saline: *F*_1, 5_ = 3.40, *P* = 0.12; Figure 1). However, there was a significant interaction between time and treatment (*F*_3,15_ = 5.01, *P* = 0.01, Figure 1), caused by a marked increase in cortisol levels 2 hours after the physiological challenge with ACTH (Planned comparisons; 2 h versus 0 h, 4 h and 24 h: all *P*≤0.01; see Figure 1).

### Responses of Captive Gobies to Predators

**Figure 2:**
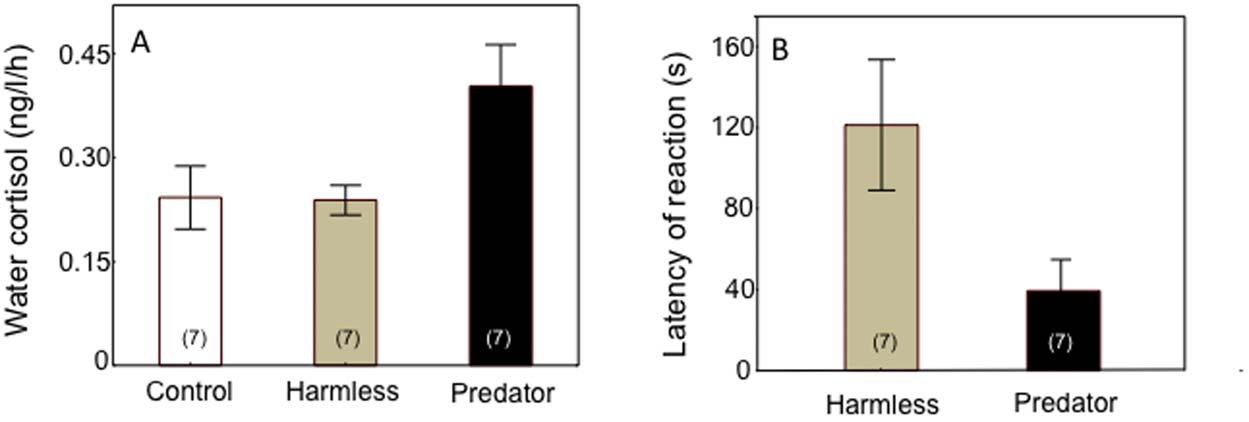
Responses of captive Caribbean cleaning gobies to exposure to a control (no fish client; white bars), harmless clients (grey bars) and predatory clients (black bars) in terms of: (a) cortisol concentration in holding-water, and (b) latency of reaction to client stimulus (i.e. time taken to move within 5 cm of client) (s). Means are shown ±1 SEM. Sample sizes are given in parentheses.

Overall, the cortisol response of cleaning gobies varied significantly across stimulus types (i.e., control, harmless or predatory fish) (1-way RM-ANOVA, *F*_2, 12_ = 6.43, *P* = 0.01; Figure 2-A). Planned comparisons revealed that cortisol level was significantly higher when cleaners were exposed to predatory stimuli than to a control stimulus (i.e., no client) (predator vs control: *F*_1,6_ = 10.16, *P* = 0.02). In contrast, cortisol levels were similar when gobies were exposed to harmless clients and to a control (harmless vs control: *F*_1,6_ = 0.01, *P* = 0.91; Figure 2-A).

The latency of cleaning gobies to react to visual stimuli was significantly lower in the presence of predators than when exposed to harmless clients (Paired t-test: *t*_6_ = 2.57, *P* = 0.04; Figure 2-B).

### Responses of Wild Gobies to Predators

**Figure 3:**
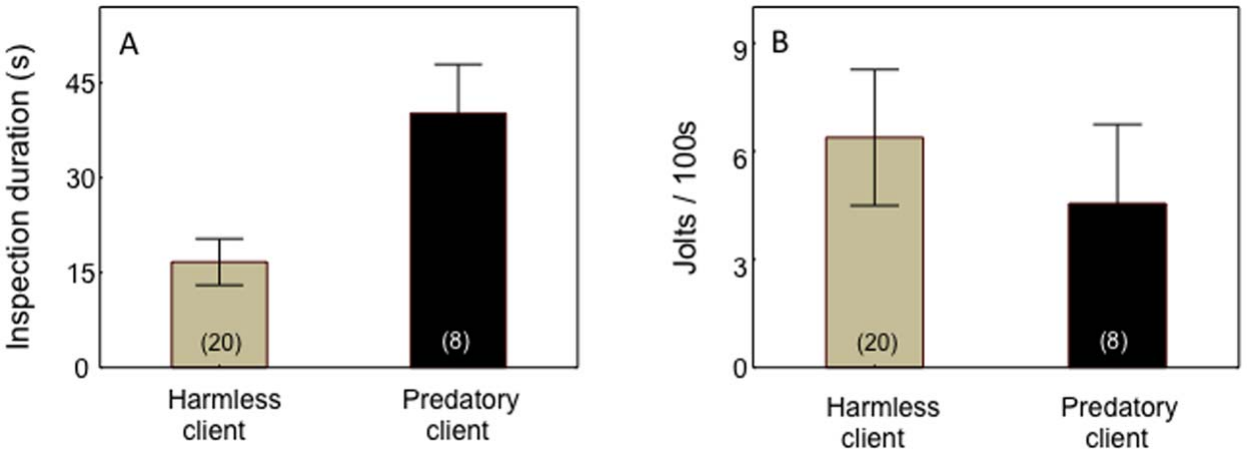
Behaviour of wild Caribbean cleaning gobies towards harmless (grey bars) and predatory clients (black bars) in terms of: (a) client inspection duration at cleaning stations (s), and (b) number of jolts by clients per 100 s of inspection. Means are shown ±1 SEM. Sample sizes ( =  number of client species) are given in parentheses.

Field observations revealed that cleaning gobies spent more time per inspection event interacting with predatory than with harmless clients (Independent samples *t* test, *t_28_* =  −3.12, *P* = 0.004; Fig. 3-A). However, the jolting rates of predatory and harmless clients were similar (*t_28_* = 0.55, *P* = 0.58; Fig. 3-B).

## Discussion

Our results provide the first physiological evidence that cleaners might perceive predators as a potential threat. This was demonstrated by the rise in relative cortisol levels of captive cleaning gobies when in visual contact with predatory stimuli. We also found that cleaners respond to the presence of dangerous clients by approaching them more swiftly (in captivity [this study], and in the wild [12]) and by cleaning them for longer (in the wild [this study]), than they do with harmless clients. Thus, the effects of predation risk seem to play an important role in mediating changes in foraging behaviour of cleaning gobies. We hypothesise that cleaners mitigate the potentially harmful physiological consequences elicited by predatory clients as aversive stressors (i.e., increase of stress levels that lead to costs in behavioural activity) by becoming increasingly proactive and by reducing the time elapsed between client approach and the start of the interaction process. The activation of this stress response may also be responsible for the prolonged cleaning service provided by these cleaners to predatory clients in the wild.

In response to an aversive stimuli (such as a predator), fish typically either trigger their inhibitory behavioural system (i.e., freezing behaviour) or activate the “flight or fight” behavioural mechanism, which enables a pro-active response [25]. The magnitude of the physiological response is usually linked to one of these coping styles [11]: freezing is usually underlined by higher levels of cortisol release, whereas fighting or fleeing is normally characterised by lower cortisol responses. The cleaning gobies in our experiment, which showed signs of pro-activity (i.e., shorter latency to react) towards predators while exhibiting higher cortisol levels, do not fit neatly into this expected dichotomy. It is possible that the stress response mechanism of our captive cleaning gobies was activated because they were forced to stay in visual contact with an aversive stimulus while being prevented from interacting with it. In contrast, in the wild, cleaning gobies can and do respond, and interact immediately with dangerous clients, thus perhaps pre-empting a rise in their cortisol levels [12]. Nevertheless, the short-term stress elicited by predators in captivity sheds some light on the behaviour of cleaning gobies in the wild.

Our field observations revealed that cleaning gobies spent longer inspecting predators than non-predatory clients, despite the fact that harmless species (e.g., parrotfishes) are relatively heavily parasitized and are among their most preferred clients [12]. Predator-induced stress may be the cause of this unexpected behavioural bias. It remains unclear whether the stress levels of cleaning gobies stay high or decrease once interactions with predators have begun. In Barbary macaques, for example, the delivery of grooming is associated with a decrease of stress levels in the groomer [26]. A possible reduction of cleaner stress levels while interacting with predatory clients could explain why cleaners do not curtail the length of their interactions with predators, as commonly occurs in *L. dimidiatus* [22], [23]. In fact, it seems likely that stress reduction during interactions with predators would occur in cleaning gobies, because of their preference for ectoparasites over other client-gleaned items such as scales and mucus [27]. The scope for conflict arising from prolonged interactions between cleaning gobies and predatory clients is therefore much reduced compared to *L. dimidiatus*, which must feed against their preference for client mucus to avoid conflict [28]. A next important research step is therefore to examine cleaner stress levels during and after interaction with clients, particularly predators, and test how fluctuations in cortisol levels (with use of higher infusions of cortisol or by blocking its effects) might produce shifts in cleaner behaviour, such as changes in client preferences or in the quality of service they provide.

Although appearing somewhat paradoxical, the behaviour of prey approaching predators has been reported in a wide variety of taxa [1]. While the costs of this behaviour are clear, benefits also exist, such as gaining information about the nature of the potential danger or even deterring a potential predator attack. In the case of cleaning mutualisms, servicing predators swiftly so that they leave sooner encourages the return of non-predatory clients to the cleaning stations [12]. Thus, the behavioural response of cleaning gobies to dangerous clients may serve as a sort of a pre-conflict management strategy [29] in which a pro-active and positive (i.e., honest) approach leads to a safe outcome for present and future interactions. In this way, cleaners first rapidly signal their presence to predatory clients and then engage immediately into cleaning, thus significantly reducing the scope for possible predatory danger or conflict.

Taken together, our results suggest that cleaning gobies may have a paradoxical way of dealing with predator-induced stress: they appear to become increasingly pro-active and prolong their cleaning investment with potentially dangerous clients. We must emphasize that our results are preliminary because our experimental design, which was by logistical necessity pseudoreplicated, permits only a restricted scope of inference in relation to the limited number of clients used. Future studies should include more client individuals and species to evaluate fully the generality of our findings. Nevertheless, we believe that our study provides initial insights into the potential endocrine mechanisms, which might underlie cleanerfish motivation to interact preferentially with some clients over others. Moreover, it adds valuable information about the potential effects of short-term stress in the regulation of cleanerfish behaviour, which have been proposed but not demonstrated for another cleaning system (the cleaner wrasse *L.* *dimidiatus*) [23], and the potential role of stress in shaping the interactive dynamics of cooperative behaviour. Future research should also focus on the physiological mechanisms responsible for the fast decision-making processes and behavioural flexibility of cleanerfish. Good candidates may be fish brain monoamines, namely serotonin, which have been associated with social responses to stressors (including predators; [30]), and the neurohormone arginine-vasotocin (the non-mammalian homologues of vasopressin), which is known for its influence in vertebrate social behaviour [31] and aggression [32], [33]. Arginine-vasotocin seems to play an important role in the mediation of interspecific cleaning behaviour of the cleaner wrasse *L. dimidiatus* [34]. Additional studies on social vertebrates as well as other cleanerfish (both obligate and facultative) and their non-cleaning relatives might reveal similarities in how individuals cope with stress caused by potential antagonists such as dominant group members or potential predators.

## Materials and Methods

### Study Species, Fish Collection and Housing Conditions

This research was conducted at the Bellairs Research Institute, Barbados. We focussed on the sharknose cleaning goby (*E.* *evelynae*), the main obligate cleanerfish species present on Barbadian fringing reefs. Seven gobies were collected from nearby fringing coral reefs 2–3 wk prior to the beginning of experiments to acclimatize to laboratory conditions. Gobies were caught with hand nets and placed individually in sealed plastic bags filled with seawater. We also captured one individual of each of four species of client fish, which were selected because these species are frequent visitors to cleaning stations and easy to keep in captivity. Two species that consume mainly benthic invertebrates (e.g., molluscs, echinoderms, cnidarians and crustaceans) were considered to be harmless clients: French Grunt (*Haemulon flavolineatum*), and Whitespotted Filefish (*Cantherhines macrocerus*); and two species were piscivorous clients: the Graysby grouper (*Cephalopholis cruentata*) and Spotted Moray (*Gymnothorax moringa*) [35]. All collected fish were initially kept singly (clients) or together (gobies) in individual glass aquaria (61 cm long · 38 cm wide · 46 cm high) with running seawater.

### Experimental Design and Behavioural Observations

To minimize the effects of previous social experience on behaviour and steroid levels, cleaning gobies were transferred to small individual aquaria (20 cm long·10 cm wide·50 cm high), which allowed visual contact with neighbouring gobies, at least 2 days before each experiment. Experiments were always carried out in the morning to avoid time-related fluctuations in cortisol levels.

Client species observed in captivity and in the wild were categorized as either potentially predatory or harmless (non-predatory) to cleaning gobies based on published diet information [35]. On each experiment day, each cleaning goby was randomly assigned to one of five clients, belonging to three exposure categories: a) predatory fish (Grasby and Spotted Moray), b) harmless fish (French Grunt and Whitespotted Filefish) and c) control (no client fish). The aquarium containing a cleaning goby was then slowly lowered into a side of a larger aquarium containing one client or no client (control). Since each cleaning goby was previously acclimatized to its smaller aquarium, this allowed both gobies and clients to be tested within their own territory. Also, this set-up allowed visual but not physical contact between goby and client. Cleaning goby behaviour was videotaped with a Sony Handycam digital videocamera (model DCR-TRV10E) placed 60 cm from the front wall of the outer aquarium, for 15 min following the introduction of the cleaner. The aquarium containing the cleaner then remained in the client or control tank for a further 45 min, to allow hormone accumulation in the water. Each goby was exposed to each of the five clients (belonging to 3 exposure categories as described above) over the course of the study. Video recordings were analysed using the software package Noldus Observer XT (Noldus Information Technology).

### Behavioural Observations in the Wild

To complement the information obtained from experimental trials with captive fish, *in situ* observations of interactions between cleaning gobies and their client fish were carried out using SCUBA. Seventy-one cleaning stations were selected haphazardly across 8 reefs on the west coast of Barbados (which included 2 of the reefs used also for goby collection for following laboratory experiments). Each cleaning station was observed once for 30 min, between 10.00 and 17.00 hours. Observations were made from a distance of 2–3 m and began after a 2- to 5-min delay to allow the fish to become accustomed to the presence of the observer. During each observation period, we recorded on plastic slates the duration(s) of inspection and the number of jolts for each visiting client. Jolts are apparent reactions to a cleanerfish bite and have previously been shown to be dishonest bites by cleaners [36], [37]. A total of 28 different client species (20 harmless and 8 predatory clients, based on published diet information [35]) were seen visiting cleaning goby stations.

### Hormone Assay

Due to the small size of cleaning gobies (max. 3.5 cm total length), we assayed steroid hormones non-invasively from fish-holding water. Holding-water steroid measurements represent a temporal integration of the cortisol levels that have been in circulation and that have been transferred to the water both by excretion (via urine and faeces) and by diffusion through the gills [38]. In order to validate this method for *E. evelynae*, individual gobies (that were previously used during experimental procedures) were injected intraperitoneally in the laboratory either with adrenocorticotropic hormone (ACTH, Sigma A-6303; 0,023 IU/g body weight) or a saline solution and then, cortisol response curves were measured from water samples. Water was exchanged at the end of each of four consecutive hours and each sample was analysed once (at the end of one hour) for cortisol content. Each sample was filtered through a C18 solid phase extraction cartridge (Merck LiChrolut RP-18, 500 mg), previously activated with 2×5 ml ethanol followed by 2×5 ml distilled water and then stored at –20°C. The adsorbed material was later eluted with 2 x 2 ml ethanol. Free and conjugated steroids (sulphates and glucuronides) were extracted and the fractions for each sample pooled and radioimmunoassayed for total cortisol as an indicator of the stress status of each individual. Cortisol assays used the commercial antibody ‘Anti-rabbit, Cortisol-3′ [ref: 20-CR50, Interchim (Fitzgerald), Montluçon, France, cross-reactivity: cortisol 100%, Prednisolone 36%, 11-Desoxycortisol 5.7%, Corticosterone 3.3%, Cortisone <0.7%] and the radioactive marker [1,2,6,7–3H] Cortisol [ref: TRK407-250mCi, Amersham Biosciences, Piscataway, NJ/USA].

### Statistical Analysis

Cortisol levels obtained from gobies in the hormone assay validation experiments were analysed using a two-way repeated measures analysis of variance (RM-ANOVA) with time (four sampling points) as a within-subject factor and treatment (ACTH versus saline) as between-subjects factor. Planned comparisons of least squares means were subsequently carried out to determine whether cortisol levels varied with treatment and across time periods.

The holding-water cortisol levels of cleaning gobies in response to visual contact to harmless, predatory fish or in the absence of stimuli (no fish) were averaged for each of these exposure categories and then analysed using a one-way RM-ANOVA with exposure category type as within-subject factor. ANOVAs were followed by planned comparisons of least squares means to test for effect of exposure. The latency to react of cleaning gobies (i.e., the time in seconds taken to move within 5 cm of the client), derived from videotapes, was averaged for each of these exposure categories (harmless and predatory fish) and then analysed by using a paired samples t-test.

From field observations, we derived two measures of cleaning service quality: (1) mean duration of inspection by cleaning gobies, and (2) the number of jolts per 100 seconds of inspection. Mean inspection durations and number of jolts were obtained for each client species across all observed cleaning events and were then compared between predatory and harmless clients using independent *t* tests.

### Experimental Design Caveat

In our laboratory experiments, we attempted to make the response variable of interest, i.e. the cortisol level of individual gobies, as independent as possible across cleaners. We did so by randomising the order of treatment presentation and fully changing the water between trials to prevent any hormonal carry-over effects. By allowing only visual contact between cleaner and client during the hormonal accumulation part of the trials, we may also have reduced the potential for non-independence which could have arisen from variation in chemical cues across clients. In addition, the repeated testing of individual cleaning gobies is acknowledged in the repeated-measures analysis. Nevertheless, because it was difficult to catch and logistically impossible to house more individual clients, our experimental design remains pseudoreplicated by the repeated use of the same individual clients across trials. Our scope of inference is therefore theoretically limited to the individual clients used (two individuals in each of two client categories). Thus, it is possible, in principle that our measured effects could be due to the state of these specific client individuals rather than to the category of risk they present to cleaning gobies. However, we consider this unlikely for two reasons. First, it is of paramount importance for cleaners to identify predators as such and to adjust their behaviour accordingly relative to non-predatory clients (cleaning gobies *Elacatinus* spp: [12]; cleaner wrasse *L. dimidiatus*: [22]; cleaner shrimp *Periclimenes longicarpus*: [39]). It thus seems unlikely that the current state of an individual client should have a greater influence on a cleaner’s physiology than how the threat it is perceived to present to that cleaner. Second, the behaviour of cleaning gobies in the laboratory corresponded well to expectations based on natural observations (see Results; [12]). We therefore believe that in spite of the inevitably pseudoreplicated experimental design, our results are representative of the general response of cleaning gobies to clients presenting contrasting levels of predation risk.

### Ethical Commitment

Ethical clearance to work at Folskestone Marine Park (Barbados, West Indies), involving animal experimentation, was obtained from the Bellairs Research Institute (McGill University, Canada) and by the Portuguese National Authority for Animal Health (Direcção Geral de Veterinária - oficio circular n° 9–0420/000/000, 20/01/2011). The use of animals and data collection complied with the laws of Barbados, Portugal and Switzerland.
